# Treatment strategy in stereotactic radiosurgery for trigeminal neuralgia, essential tremor, and coexisting intracranial tumors: The impact of biologically effective dose on clinical outcome

**DOI:** 10.1002/acm2.70436

**Published:** 2025-12-29

**Authors:** Sarthak Sinha, Victor Goulenko, Venkatesh Shankar Madhugiri, Shefalika Prasad, Neil D. Almeida, Rohil Shekher, Matthew B. Podgorsak, Robert J. Plunkett, Dheerendra Prasad

**Affiliations:** ^1^ Department of Radiation Medicine Roswell Park Comprehensive Cancer Center Buffalo New York USA; ^2^ Department of Neurosurgery Roswell Park Comprehensive Cancer Center Buffalo New York USA; ^3^ Department of Radiation Medicine and Neurosurgery Jacobs School of Medicine and Biomedical Sciences Buffalo New York USA

**Keywords:** biologically effective dose, dual‐target treatment, essential tremor, functional radiosurgery, gamma knife radiosurgery, intracranial tumor, stereotactic radiosurgery, trigeminal neuralgia

## Abstract

**Background:**

Gamma Knife radiosurgery (GKRS) is a well‐established treatment for trigeminal neuralgia (TN) and essential tremor (ET). In patients with coexisting intracranial tumors, radiosurgery can potentially address functional and oncologic targets in a single or staged session. However, data on integrating these treatments and the predictive role of the biologically effective dose (BED), remain limited. This study aims to evaluate clinical outcomes and identify the influence of dosimetric predictors, including BED, in patients undergoing GKRS for TN and/or ET in the context of coexisting intracranial tumors.

**Methods:**

This retrospective analysis included 12 patients treated with GKRS for TN and a coexisting intracranial tumor, and two patients treated for ET and a tumor. Clinical outcomes were assessed using Barrow Neurological Institute (BNI) pain and numbness scores. Treatment parameters, including prescribed dose, dose rate, and BED, were analyzed. BED thresholds for response prediction were identified using logistic regression and receiver operating characteristic (ROC) analysis.

**Results:**

Clinically meaningful pain relief was observed in 76.5% of all GKRS treatment sessions, including instances where patients underwent repeat GKRS. Among 12 analyzable TN patients (15 treatment sessions), five underwent repeat GKRS for recurrent or persistent pain. Of the patients with available imaging, 50% showed tumor shrinkage, while the remainder were radiological non‐responders; two of these three patients were among the five who required repeat GKRS. Repeat treatments were well‐tolerated, with no increase in complications or radiation necrosis. BED was significantly associated with early BNI improvement across the cohort (Spearman's *ρ* = 0.660, *p* = 0.0054; Pearson's *r* = 0.718, *p* = 0.0017, R^2^ = 0.515) and even more strongly in patients with tumor‐related TN (*ρ* = 0.797, *p* = 0.01). ROC analysis identified BED thresholds predictive of early responders: 1544.9 Gy_2_._47_ for the full cohort (AUC = 0.78) and 1478.71 Gy_2_._47_ for the tumor‐compression subgroup (AUC = 0.85). Tertile‐based BED stratification showed significant differences in pain relief in the tumor‐compression group (*p* = 0.05), but not in the full cohort.

**Conclusion:**

GKRS is safe and effective for TN in patients with intracranial tumors. BED appears to be a valuable predictor of early treatment response, particularly in tumor‐related TN, where it demonstrated enhanced predictive strength. These findings support integrating BED into treatment planning and highlight the broader utility of radiosurgery in addressing coexisting functional and oncologic pathologies. Prospective studies are warranted to validate these observations and optimize dose‐guided strategies.

## 1 INTRODUCTION

Trigeminal neuralgia (TN) is a neuropathic condition characterized by recurrent, brief episodes of severe, electric‐shock‐like pain distributed along one or more branches of the fifth cranial nerve, most often affecting the maxilla, forehead, or mandible.^1^ While the majority is attributed to vascular compression, commonly by the superior cerebellar artery, tumor‐related trigeminal neuralgia (TRTN) accounts for up to 1%–13% of presentations.^2^, ^3^, ^4^ When feasible, open surgical resection of compressive lesions is considered the treatment of choice for TRTN, offering the possibility of both tumor control and pain relief.^5^, ^6^, ^7^ However, in patients unfit for surgery, stereotactic radiosurgery (SRS) has emerged as an effective and minimally invasive alternative.^8^, ^9^, ^10^, ^11^, ^12^, ^13^, ^14^

Traditional SRS strategies in TRTN have focused on tumor targeting to relieve nerve compression,^9^, ^10^, ^14^ whereas idiopathic TN is typically managed through irradiation of the trigeminal root entry zone (TREZ),^9^ or its preganglionic zone. Several studies have shown that targeting only the tumor in TRTN may be associated with higher recurrence (24%–47%) and increased complications such as facial numbness.^15^, ^16^, ^17^ In contrast, dual‐target approaches, irradiating both the tumor and the TREZ, have demonstrated promising results in terms of initial pain control and durability without increasing treatment‐related toxicity.^16^, ^18^ Beyond TRTN, patients often present with intracranial tumors unrelated to their TN symptoms. In such scenarios, SRS offers an opportunity to treat both entities, regardless of causal relationship, through either a simultaneous or staged approach.

Additionally, SRS has been effective in managing other functional disorders, such as essential tremor (ET), which affects up to 1% of the population and can drastically impair quality of life.^19^, ^20^ Gamma Knife thalamotomy has been validated as a safe and effective treatment modality for ET, particularly in patients with contraindications to deep brain stimulation.^8^, ^20^, ^21^, ^22^, ^23^, ^24^

Several studies have reported a dose‐response relationship between radiation delivery to the trigeminal nerve and clinical outcomes in TN, emphasizing the importance of optimizing biological dosing.^25^, ^26^ However, limited data exist on how dose modulation affects outcomes when treating TN concurrently with an intracranial mass. Similarly, to the best of our knowledge, the simultaneous radiosurgical management of ET and intracranial tumors has not been previously described in the literature.

In this retrospective study, we evaluated outcomes in patients who underwent SRS for TN and/or ET in the setting of intracranial tumors. Specifically, we aimed to: access pain relief and complication rates in patients receiving either simultaneous or staged treatment of TN and tumor; investigate the influence of biologically effective dose (BED) on short‐term clinical response and its predictive utility, and explore the feasibility, safety, and functional outcomes of patients undergoing combined SRS for ET and intracranial tumors. By analyzing clinical response in this dual‐pathology cohort, we aim to provide insight into the safe and effective integration of functional and oncologic radiosurgery planning in complex neurosurgical presentations. Through this broader lens, we aim to contribute to the evolving paradigm of precision‐targeted radiosurgery, where the art lies not only in treating disease but in restoring function with finesse.

## 2 METHODS

Of the 250 patients treated for TN with SRS between 1998 and 2023 at our institution, we retrospectively identified a subset of patients who had been treated for both a diagnosed intracranial tumor and TN. Additionally, we included patients who were treated for ET in combination with an intracranial tumor, regardless of whether the treatments were performed in a single session or across multiple sessions.

### 2.1 Inclusion criteria

Patients were included in the study if they met the following criteria: (1) diagnosis of TN, defined as recurrent, intense, sudden, shock‐like, or stabbing episodic facial pain occurring along one or more divisions of the trigeminal nerve, consistent with diagnostic criteria,^27^ that underwent Gamma Knife radiosurgery (GKRS) targeting the TN between 1998 and 2023; (2) diagnosis of essential tremor, defined as an action tremor affecting the upper limbs, head, face/jaw, voice, tongue, trunk, or lower limbs, without other neurological signs, and received GKRS thalamotomy for ET during the same period; and (3) radiologically confirmed intracranial mass (arteriovenous malformations [AVM] and tumor) treated with GKRS during 1998 to 2023, either concurrently (in the same session) or sequentially (in separate sessions) with TN or ET treatment.

This study included 12 unique patients with TN and two patients with ET, all of whom also had intracranial tumors. The 12 TN patients underwent 17 functional GKRS sessions: 12 initial sessions and five repeat sessions (denoted by a prime symbol in Tables 1 and 2). A “patient” refers to a unique individual; a “session” refers to a single GKRS treatment delivery; a “repeat session” indicates a subsequent functional GKRS for recurrent or persistent symptoms; a “functional treatment” refers to GKRS targeting the trigeminal nerve or thalamus; and a “tumor treatment” refers to GKRS targeting the coexisting intracranial lesion. Some patients underwent both tumor and functional GKRS, either sequentially or as staged treatments, but each treatment delivery was counted as a separate session.

**TABLE 1:** Patient characteristics for all trigeminal neuralgia (TN) patients treated with Gamma Knife radiosurgery (GKRS).

Patient ID	Session	Sex	Age (years)	Tumor type	Tumor side and TN side	Dose to the tumor (Gy)	Dose for TN (Gy)	Cumulative dose (Gy)	TRTN	Interval between tumor and TN treatment (months)	BED (Gy_2_․_47_)	BNI pre‐treatment	BNI 1st, 2nd, and 3rd FU (time to follow‐up [months])	Δ total BNI	Complications
1	Single	M	50	AVM	Right middle cerebellar peduncle; right TN	22	70	105.9	Yes	7	1434.02	V	V (6 m), V (14 m), V (20 m)	0, 0, 0	–
2	Single	F	74	Vestibular schwannoma	Right VS extending to the right IAC; right TN	11	60	60	Yes	0	1151.48	V	IV (0.4 m), IV (3 m), 4 (10 m)	0, 0, −2	Numbness
3	Initial	M	48	Vestibular schwannoma	Right IAC, left TN	12	80	80	No	139	1955.56	V	II (1 m), IV (4 m)	−4, −1	–
3’	Repeat	Same as above					50	82.7	No	7 m from previous GKRS	846.36	IV	V (1 m), IIIb (3 m)	+1, −1	Numbness
4	Single	F	53	Metastasis	Left cerebellar region, right TN	20	80	80.10	No	7	1999.99	V	IV (1 m), IIIa (2 m), II (5 m)	−1, −3, −4	–
5	Single	M	90	Meningioma	1‐ Left frontal para sagittal meningioma, 2‐ Right smaller falx meningioma; Left TN	12	80	80	No	5	2026.68	V	II (2 m), IIIa (3 m), IIIb (4 m)	−3, −2, −2	–
6	Initial	F	49	Meningioma	1‐ Right cavernous sinus, 2‐Right middle cranial fossa, involving pre pontine cistern abutting right anterolateral part of pons; Right TN	13	80	89.3	Yes	65	1837.83	V	IV (2 m), IV (3 m), V (4 m)	−1, −1, 0	–
6’	Repeat	Same as above					60	137.7	Yes	11 m from previous GKRS	1111.88	V	V (2 m), IV (3 m), IIIb (4 m)	0, −1, −2	–
7	Initial	M	61	Meningioma	Left prepontine; left TN	12	80	106.3	Yes	39	1872.93	V	II (2 m), IIIa (5 m), V (12 m)	−4, −3, 0	–
7’	Repeat	Same as above					70	176.3	Yes	39 m from previous GKRS	1523.39	V	IIIb (1 m), IIIb (8 m), IIIb (20 m)	−2, −2, −2	Numbness
8	Initial	F	49	Meningioma	Tuberculum sella; left TN	10	80	80	No	109	1994.94	V	IIIb (1 m), IV (4 m), V (6 m)	−2, −1, 0	–
8’	Repeat	Same as above					70	133.2	No	39 m from previous GKRS	1607.75	V	IIIa (3 m), IIIa (3 m), II (10 m)	−3, −3, −4	–
9	Single	F	63	Metastasis	Right temporal lobe, involving right middle fossa, medial extension into sphenoid sinus and Meckel's cave; right TN	16	70	76.1	Yes	2	1544.90	V	IIIa (1 m), IIIa (2 m)	−3, −3	–
10	Single	F	51	Pituitary adenoma	Pituitary adenoma; right TN	15	70	80.3	No	11	1556.85	V	IIIb (1 m), IIIb (5 m), IIIb (12 m)	−2, −2, −2	–
11	Single	F	81	Meningioma	Right CPA, tentorial meningioma; right TN	10	80	87.8	Yes	60	2092.73	V	II (0.1 m), V (1 m)	−4, 0	–
12	Initial	F	83	Vestibular schwannoma	Left IAC; left TN	12	70	77.8	Yes	14	1596.08	V	IIIb (1 m), IIIb (4 m), V (7 m)	−2, −2, 0	–
12’	Repeat	Same as above					70	147.8	Yes	37 m from previous GKRS	–	V	IIIa (1 m), IIIa (3 m), II (10 m)	−3, −3, −4	–

*Note*: Each row represents an individual GKRS treatment session; repeat sessions are indicated by a prime symbol (′) following the patient ID. “Single” refers to patients who underwent one functional GKRS procedure, while “Initial” and “Repeat” indicate first and subsequent GKRS treatments, respectively. “Interval between tumor and TN treatment” refers to the time between oncologic and functional GKRS sessions when performed separately. “Complications” lists new or worsened sensory changes reported after GKRS.

Abbreviations: AVM, arteriovenous malformation; BED, biologically effective dose (Gy_2_․_47_, calculated with *α*/*β* = 2.47 Gy); BNI, Barrow Neurological Institute pain intensity score; CPA, cerebellopontine angle; F, female; FU, follow‐up; GKRS, Gamma Knife radiosurgery; IAC, internal auditory canal; M, male; m, months; TN, trigeminal neuralgia; TRTN, tumor‐related trigeminal neuralgia (radiological contact or compression of the trigeminal root entry zone by the tumor); VS, vestibular schwannoma; Δ BNI, change in BNI score from baseline.

**TABLE 2:** Patient characteristics for tumor‐related trigeminal neuralgia (TRTN) subgroup.

Patient ID	Session	Sex	Age (years)	Tumor type	Tumor side and TN side	Dose to the tumor (Gy)	Dose for TN (Gy)	Cumulative dose (Gy)	TRTN	Interval between tumor and TN treatment (months)	BED (Gy_2_․_47_)	BNI pre‐treatment	BNI 1st, 2nd, and 3rd FU (time to follow‐up [months])	Δ total BNI	Complications
1	Single	M	50	AVM	Right middle cerebellar peduncle; right TN	22	70	105.9	Yes	7	1434.02	V	V (6 m), V (14 m), V (20 m)	0, 0, 0	–
2	Single	F	74	Vestibular schwannoma	Right VS extending to the right IAC; right TN	11	60	60	Yes	0	1151.48	V	IV (0.4 m), IV (3 m), IIIb (10 m)	0, 0, −2	Numbness
6	Initial	F	49	Meningioma	1‐ Right cavernous sinus, 2‐Right middle cranial fossa, involving pre pontine cistern abutting right anterolateral part of pons; right TN	13	80	89.3	Yes	65	1837.83	V	IV (2 m), IV (3 m), V (4 m)	−1, −1, 0	–
6’	Repeat	Same as above					60	137.7	Yes	11 m from previous GKRS	1111.88	V	V (2 m), IV (3 m), IIIb (4 m)	0, −1, −2	–
7	Initial	M	61	Meningioma	Left prepontine; left TN	12	80	106.3	Yes	39	1872.93	V	II (2 m), IIIa (5 m), V (12 m)	−4, −3, 0	–
7’	Repeat	Same as above					70	176.3	Yes	39 m from previous GKRS	1523.39	V	IIIb (1 m), IIIb (8 m), IIIb (20 m)	−2, −2, −2	Numbness
9	Single	F	63	Metastasis	Right temporal lobe, involving right middle fossa, medial extension into sphenoid sinus and Meckel's cave; Right TN	16	70	76.1	Yes	2	1544.90	V	IIIa (1 m), IIIa (2 m)	−3, −3	–
11	Single	F	81	Meningioma	Right CPA, tentorial meningioma; right TN	10	80	87.8	Yes	60	2092.73	V	II (0.1 m), V (1 m)	−4, 0	–
12	Initial	F	83	Vestibular schwannoma	Left IAC; left TN	12	70	77.8	Yes	14	1596.08	V	IIIb (1 m), IIIb (4 m), V (7 m)	−2, −2, 0	–
12’	Repeat	Same as above					70	147.8	Yes	37 m from previous GKRS	–	V	IIIa (1 m), IIIa (3 m), II (10 m)	−3, −3, −4	–

*Note*: Subset of patients in whom trigeminal neuralgia resulted from direct tumor compression of the trigeminal root entry zone. Each row represents an individual GKRS treatment session; repeat sessions are indicated by a prime symbol (′) following the patient ID. “Single” refers to patients who underwent one functional GKRS procedure, while “Initial” and “Repeat” indicate first and subsequent GKRS treatments, respectively. “Interval between tumor and TN treatment” refers to the time between oncologic and functional GKRS sessions when performed separately. “Complications” lists new or worsened sensory changes reported after GKRS.

Abbreviations: AVM, arteriovenous malformation; BED, biologically effective dose (Gy_2_․_47_, calculated with *α*/*β* = 2.47 Gy); BNI, Barrow Neurological Institute pain intensity score; CPA, cerebellopontine angle; F, female; FU, follow‐up; GKRS, Gamma Knife radiosurgery; IAC, internal auditory canal; M, male; m, months; TN, trigeminal neuralgia; TRTN, tumor‐related trigeminal neuralgia (radiological contact or compression of the trigeminal root entry zone by the tumor); VS, vestibular schwannoma; Δ BNI, change in BNI score from baseline.

Of the 17 functional GKRS sessions, 16 sessions had complete dosimetric information to allow calculation of BED, and 17 sessions had at least one clinical follow‐up visit. The final evaluable cohort consisted of 16 functional sessions with complete BED and follow‐up data. All analyses were performed at the session‐level, with repeat sessions analyzed independently.

### 2.2 Exclusion criteria

Patients were excluded from the analysis if (1) follow‐up clinical data were unavailable, precluding assessment of treatment response, or (2) treatment planning parameters (including dosimetric values such as maximum dose and delivery time), were missing from the records.

### 2.3 Patient identification and data collection

Patients were identified from our institutional GKRS database following approval from the Institutional Review Board, and appropriate written consents were obtained prior to this study. A retrospective review of medical records was conducted to collect demographic details, tumor characteristics, dosimetric parameters, and clinical outcomes. GKRS treatment plans were reviewed to extract dosimetric data, including prescribed dose, maximum point dose, dose rate, and BED. The BED was calculated using the linear‐quadratic model incorporating the delivery time of each GKRS session, using previously published repair‐corrected models described by Hopewell et al.^28^ and Tuleasca et al.^29^ An *α*/*β* value of 2.47 Gy was assumed, consistent with prior reports in TN radiosurgery, reflecting the low *α*/*β* characteristics of neural tissue.^29^ (Supplement 1) For patients who underwent sequential GKRS sessions for TN, each session was analyzed independently, and cumulative BED was calculated as the sum of individual session contributions. The BEDs of the tumor treatments were not included in this analysis due to significant data heterogeneity, which could mislead the results and prevent them from accurately reflecting the impact on the clinical outcomes response. The biological recovery occurring between separate treatment courses (e.g., between a tumor session and a later functional session, or between repeat functional sessions) was not modeled and is beyond the scope of this analysis. Three patients were excluded from the analysis due to an absence of clinical follow‐up, leaving 12 analyzable TN patients.

For patients undergoing sequential treatments, each session was analyzed independently, and the cumulative dose delivered to the trigeminal nerve was calculated by adding the TN prescribed dose to the point dose from the previous treatment at the trigeminal nerve, matching the new treatment isocenter. MRI scans were used to localize intracranial tumors and identify the TREZ. Although we attempted to qualitatively compare pre‐ and post‐treatment imaging in patients where tumor abutment of the trigeminal nerve was believed to contribute to their functional symptoms, formal volumetric analysis of tumor response was not performed, as tumor control was not a primary objective of this study.

### 2.4 Patient cohort

We identified a total of 15 patients who had undergone SRS for both TN and an intracranial tumor. These included six meningiomas, four vestibular schwannomas, two brain metastases, one pituitary adenoma, one trigeminal schwannoma, and one AVM. Three patients (one with a meningioma, one with a vestibular schwannoma, and one with a trigeminal schwannoma) had no available clinical follow‐up data and were excluded from outcome analysis. Of the remaining 12 patients, five required a second GKRS session for persistent or recurrent TN symptoms.

Five patients underwent repeat GKRS for persistent or recurrent TN, resulting in a total of 17 treatment sessions analyzed. All repeats were included as independent data points for dosimetric and outcome analyses.

We also identified two additional patients who underwent GKRS thalamotomy for ET along with radiosurgical treatment of an intracranial tumor. One had a vestibular schwannoma and the other a meningioma. One patient had a sequential bilateral thalamotomy.

### 2.5 Radiosurgical technique

All functional treatments had a frame‐based GKRS. Different platforms were used in this period (Models C, Perfexion, Icon, and Esprit; Elekta AB, Sweden).

The treatment planning utilized T1‐weighted MRI with and without contrast, T2 sequences, and a CISS sequence for visualization of the trigeminal nerve. A 4‐mm collimator was used to deliver a single isocenter to the cisternal segment of the trigeminal nerve. The 35 Gy isodose line was positioned adjacent to the brainstem, while the point dose delivered ranged from 50 to 80 Gy to the 100% isodose line. Depending on the distortion of the nerve caused by the tumor, and its adequate visibility, the isocenter could be placed closer to the gasserian ganglion.

For ET, a combination of indirect targeting based on anatomical coordinates and atlas, and DTI‐tractography with visualization of the cerebello‐thalamic‐cortical tract, was used to localize the ideal target within the ventral intermediate nucleus (VIM). A 4‐mm collimator was used to deliver 120–130 Gy to the 100% isodose line.

### 2.6 Clinical assessment and outcome measures

Patient records were retrospectively analyzed to document the onset of symptoms and initial severity of TN, measured using the modified Barrow Neurological Institute (BNI) pain intensity^30^ scale: I‐ No pain without medication, II‐ Occasional pain, not requiring medication, IIIa‐ No pain, continued medication, IIIb‐ Persistent pain, adequately controlled with medication, IV‐ Some pain, not adequately controlled with medication, and V‐ Severe pain or no relief.

For each patient, we recorded the interval from symptom onset to treatment, initial BNI score, and presenting symptoms. Follow‐up assessments were based on medical record documentation of clinical reviews or telephonic interviews. At each follow‐up, pain response and treatment‐related complications were recorded, and BNI scores reassessed. Any reduction in BNI score from baseline was considered pain improvement.^31^ Failure of pain control was defined as a persistent BNI score of IV or V without improvement. Pain recurrence was defined as an increase in maximum pain following an initial improvement.^31^ Patients were classified as responders if they demonstrated a ≥ 2‐point improvement in BNI score at the first follow‐up for the calculation of optimal BED threshold.

Repeat GKRS sessions were treated as separate data points in the correlation and regression analyses, as each represented an independent treatment exposure with distinct dosimetric parameters and follow‐up outcomes. For receiver operating characteristic (ROC) and linear regression modeling each session was analyzed individually based on its corresponding BED and BNI change at first follow‐up.

Facial sensory complications were evaluated using the BNI facial numbness score: I‐ No numbness, II‐ Mild numbness, not bothersome, III‐ Numbness, somewhat bothersome, and IV‐ Numbness, very bothersome. Hypoesthesia was considered a potential complication related to radiation exposure to the brainstem and TREZ.^15^, ^16^, ^17^

Pretreatment data were missing for one patient, and post‐treatment data were unavailable for two others. While dosimetric information and imaging were retrieved for these patients, clinical outcomes were not evaluated, and they were excluded from the final outcome analysis.

### 2.7 Statistical analysis

The statistical analyses were performed with IBM SPSS Statistics software version 21.0 (SPSS IBM). A *p*‐value ≤ 0.05 was considered statistically significant.

## 3 RESULTS

A total of 15 patients who underwent GKRS for both an intracranial tumor and TN‐related pain were initially identified. Of these, three patients were excluded due to unavailable follow‐up clinical data or missing treatment parameters, resulting in a final analytic cohort of 12 patients. Baseline demographic and treatment characteristics for all TN patients are summarized in Table 1.

The cohort included eight females and four males, with a mean age of 62.7 years (range: 48–90 years) at the time of tumor treatment. The most frequently diagnosed tumors were meningiomas (*n* = 5) and vestibular schwannomas (*n* = 3), followed by brain metastases (*n* = 2), pituitary adenoma (*n* = 1), and AVM (*n* = 1). Among these 12 patients, 5 (41.6%) required a second GKRS session for TN due to persistent or recurrent pain, bringing the total number of radiosurgical treatments analyzed to 17 sessions. Of the three radiological non‐responders, two patients required repeat treatment. All repeat treatments were included in the statistical analysis as independent sessions, while still being traceable to the same patient. Among the 12 patients included in the analysis, seven had TN attributable to tumor‐related compression. Post‐treatment MRI scans were unavailable for two of these patients, and therefore, tumor response could not be assessed in these patients. For the remaining six patients, the first follow‐up MRI was available at a mean of 4 months post‐treatment (range: 2–11 months; SD = 3.16). Radiological assessment showed a reduction in tumor size in three patients (50%), while the other three (50%) demonstrated no apparent change in tumor dimensions. Notably, two of the three radiological non‐responders required repeat GKRS due to persistent TN pain. The third non‐responder had been treated for a vestibular schwannoma.

At baseline, all patients reported severe TN pain. According to the BNI pain scale, 10 patients (83.3%) had a score of V, and two patients (16.7%) had a score of IV, indicating pain not adequately controlled with medication. Only one patient received simultaneous treatment for TN and vestibular schwannoma within the same GKRS session; the remaining treatments were performed sequentially.

The radiation dose delivered to the TREZ ranged from 50 to 80 Gy, with 70 Gy being the most frequently prescribed dose. Among patients who underwent repeat treatment, the initial session consistently involved 80 Gy, while the second session employed reduced doses ranging from 50 to 70 Gy. Follow‐up data were collected for patients at multiple time points. The mean time to first follow‐up was 1.5 months (SD = 1.3 months), with the second follow‐up occurring at 4.5 months (SD = 3.1 months), and the third at 9.3 months (SD = 5.9 months).

For analysis, the cohort was divided into two groups: patients who received a single GKRS session and those who required repeat treatment.

### 3.1 Single GKRS session

Seven patients underwent a single GKRS session targeting TN. This group included two males and five females, with a mean age at tumor treatment of 66 years (range: 50–90). Tumor types included vestibular schwannoma (*n* = 1), meningioma (*n* = 2), brain metastasis (*n* = 2), AVM (*n* = 1), and pituitary adenoma (*n* = 1). Six of the lesions were ipsilateral to the side of TN pain, and in five patients, the pain was attributable to trigeminal nerve compression. Only one patient received simultaneous treatment to both the tumor and the TREZ; all other treatments were performed in separate sessions. The median interval between tumor and TN treatment was 7 months (range: 0–60 months).

Tumors in this group were irradiated with a mean dose of 15.1 Gy (SD = 4.5 Gy), at an average isodose line of 49%. The TREZ was treated with a mean dose of 72.8 Gy (range: 60–80 Gy, SD = 7.5 Gy), at a mean dose rate of 2.79 Gy/min (range: 1.9–3.3 Gy/min, SD = 0.46). The cumulative maximum point dose received an average dose of 81.46 Gy (range: 60–105.9 Gy; 95% CI: 68–94).

Six of seven patients (85.7%) had a BNI score of V before treatment, and one had a score of IV. At first follow‐up, five of seven patients (71.4%) showed improvement in BNI score. Spearman correlation analysis revealed no statistically significant association between BNI score change at first, second, or third follow‐up and tumor dose, percentage coverage, prescribed dose to the TREZ, or maximum dose point (all *p* > 0.05). The mean time to first follow‐up was 1.5 months, second follow‐up at 4 months, and third at 8 months.

### 3.2 Repeat GKRS sessions

Five patients underwent repeat GKRS due to inadequate pain control. The median interval between the two sessions was 37 months. As expected, these repeat patients received significantly higher cumulative doses than single‐session patients (mean cumulative dose: 136 Gy vs. 81 Gy; Welch's *t*‐test, *p* = 0.00025). However, neither pain improvement nor the incidence of numbness was significantly associated with the cumulative dose (Pearson correlation for BNI change, *r* = 0.18, *p* = 0.58; *t*‐test for numbness, *p* = 0.81).

Although no independent dosimetric predictors of pain relief or complications were identified, repeat treatment was associated with numerically greater BNI improvement (mean change: 2.6 vs. 1.86 in the single‐session group), albeit not statistically significant (Welch's *t*‐test, *p* = 0.35).

**FIGURE 1:**
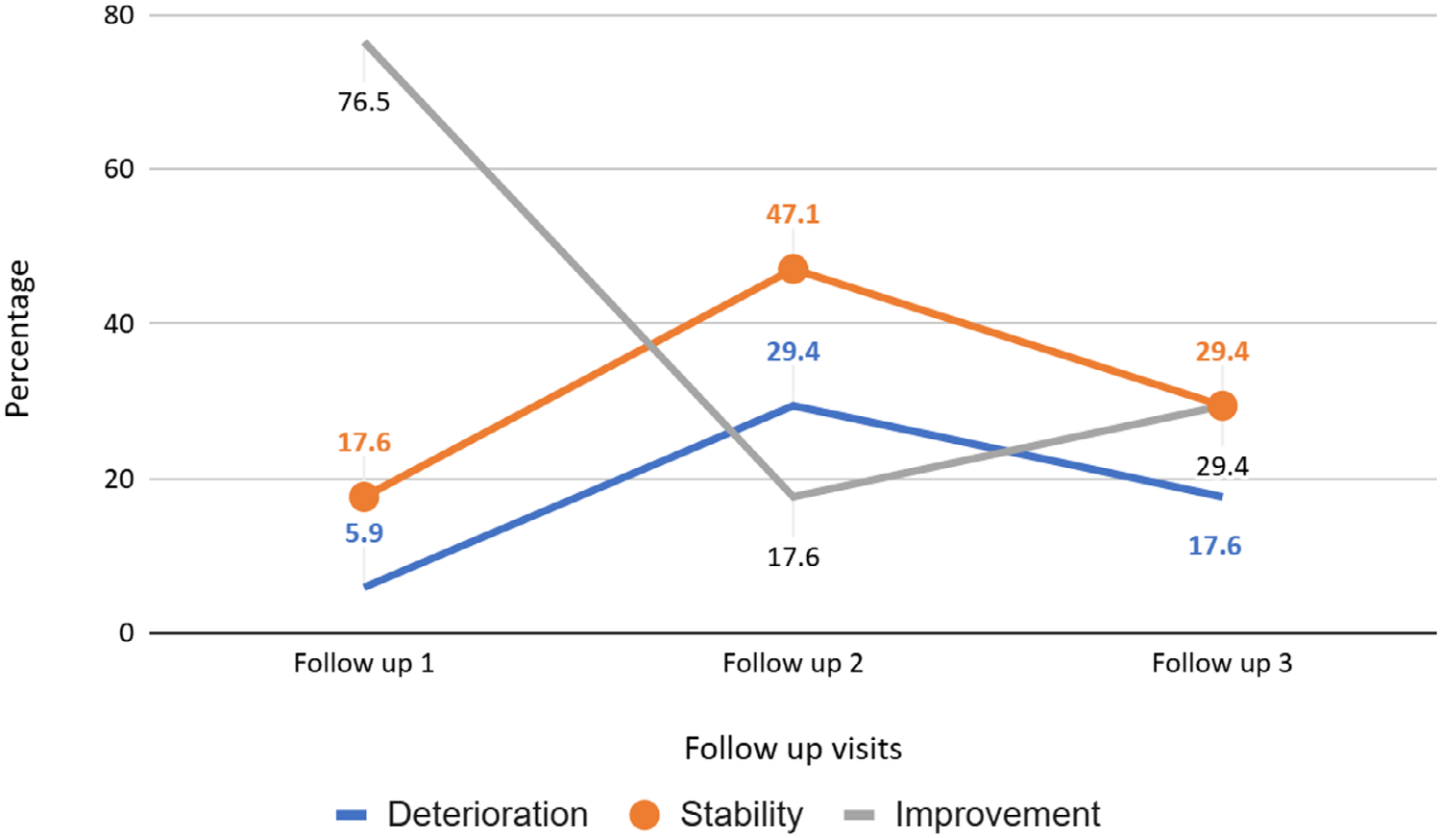
Line graph illustrating the percentage of patients experiencing improvement, stability, or deterioration in Barrow Neurological Institute (BNI) scores across consecutive follow‐up visits. Improvement: Reduction of the BNI score. Stability: BNI score equal to the pre‐treatment. Deterioration: Increase of the BNI score. The mean time to first follow‐up was 1.5 months, to the second follow‐up was 4 months, and to the third was 8 months.

Across the 17 treatment sessions (including initial and repeat GKRS procedures), clinical response at first follow‐up showed improvement in 13 patients (76.5%), stable pain in three patients (17.6%), and worsening symptoms in one patient (5.9%). BNI score changes across all follow‐ups were not significantly correlated with the functional maximum dose delivered (Spearman correlation, all *p* > 0.05). Figure 1 illustrates the distribution of clinical outcomes, including improvement, stability, and deterioration.

Patients with stable pain scores‐maintained relief for an average of 8.8 months (SD = 6.2), whereas those with worsening scores experienced relief for approximately 3.4 months (SD = 1.9). No statistically significant dependence was observed between stability duration or recurrence and treatment dose parameters.

### 3.3 Analysis of BED in relation to clinical response

A significant positive correlation was observed between BED and improvement in BNI score at the first follow‐up, indicating that higher BED values were associated with greater early pain relief. Specifically, Spearman's *ρ* was 0.660 (*p* = 0.0054) and Pearson's *r* was 0.718 (*p* = 0.0017). Since the BED values were confirmed to be normally distributed, the Pearson correlation was regarded valid. In contrast, no significant correlation was found between BED and BNI improvement at subsequent follow‐ups (second and third visits; *p* = 0.50 and *p* = 0.60, respectively), indicating that BED might be more predictive of early treatment response than of long‐term outcomes.

**FIGURE 2:**
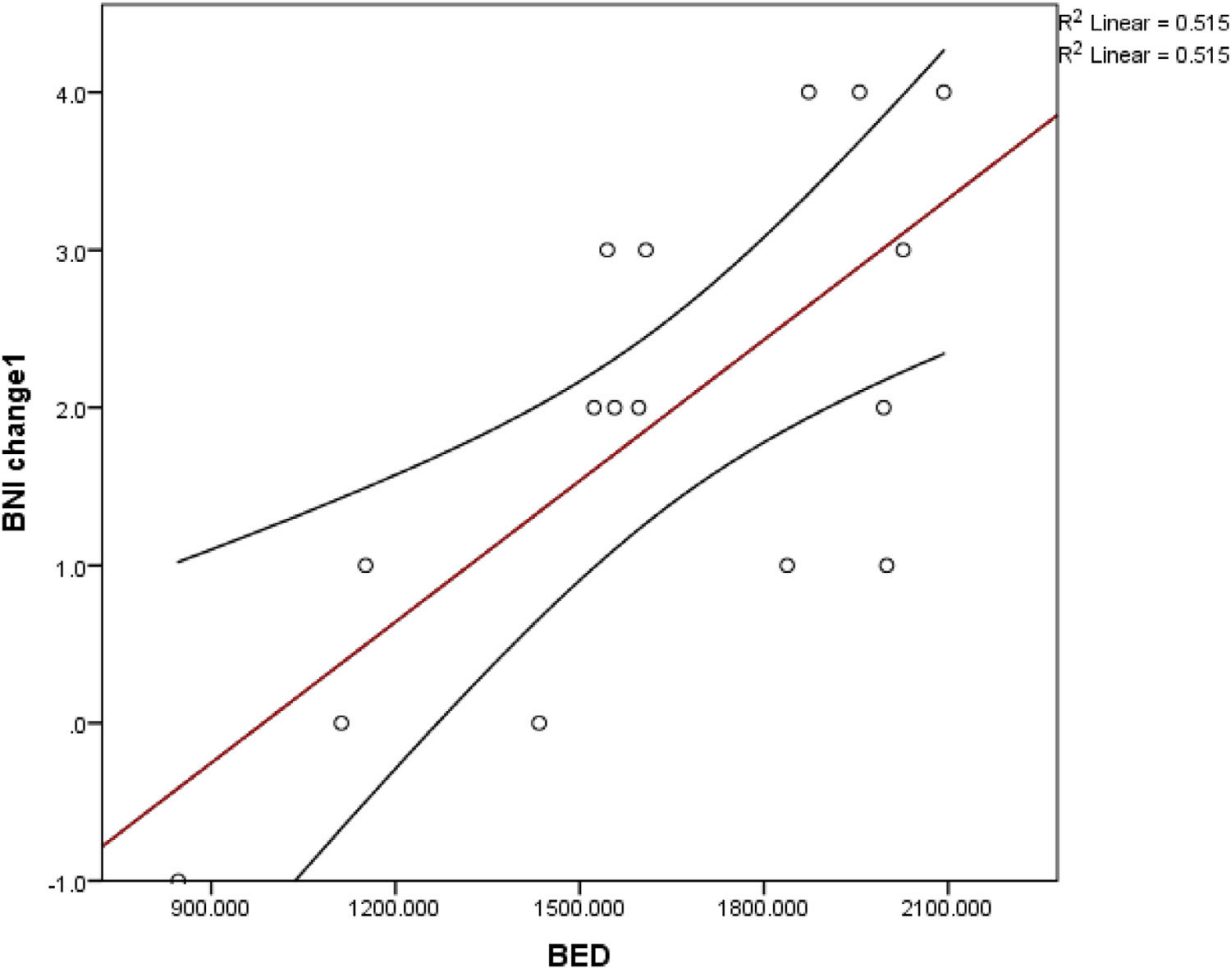
Scatterplot showing the relationship between biologically effective dose (BED) and Barrow Neurological Institute (BNI) score improvement at first follow‐up (BNI change 1). Each point represents an individual treatment session. The red regression line represents the best linear fit to the data, indicating a positive correlation between BED and improvement in BNI score (R^2^ = 0.515, *p* = 0.002). Higher BED values were associated with greater reductions in pain intensity, as measured by the BNI score. Some data points represent repeat Gamma Knife radiosurgery (GKRS) sessions for the same patient, consistent with the prime notation in Table 1.

Consistent with this, a linear regression model showed that BED was a statistically significant predictor of pain improvement at the first follow‐up (*p* = 0.0017), but not at later time points. This relationship is displayed in Figure 2, which illustrates the linear association between BED and initial BNI score change.

Furthermore, patients were classified as responders if they showed a ≥ 2‐point improvement in BNI score at the first follow‐up. Binary logistic regression using BED as the predictor indicated a borderline statistically significant association with responder status (*p* = 0.072), with the model demonstrating good discriminatory power (AUC = 0.78).

**FIGURE 3:**
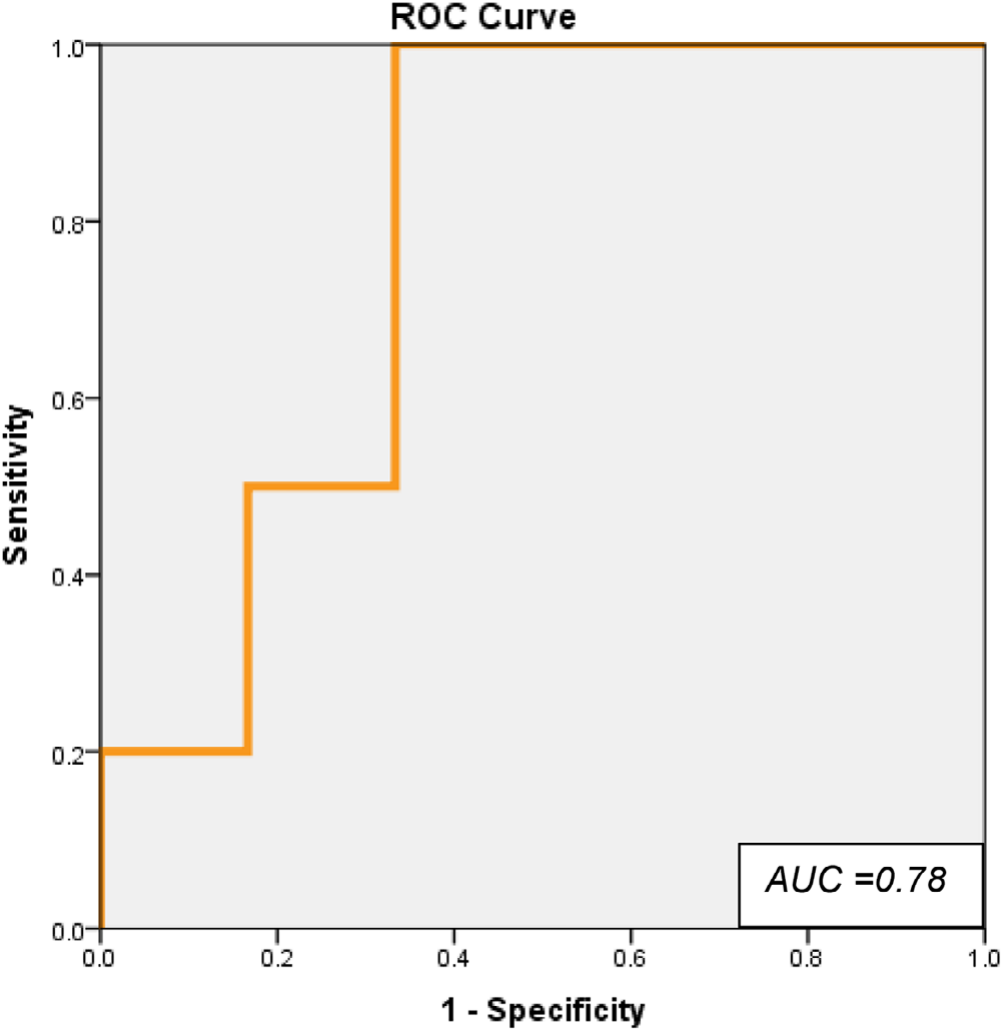
Receiver operating characteristic (ROC) curve illustrating the predictive performance of biologically effective dose (BED) for identifying responders (≥ 2‐point BNI score improvement at first follow‐up). The model yielded an AUC of 0.78. The optimal BED threshold, determined using Youden's Index, corresponds to a predicted probability cutoff of 0.573 (BED = 1544.9 Gy_2.47_), achieving 100% sensitivity and 67% specificity.

The ROC curve (Figure 3) illustrated a favorable trade‐off between sensitivity and specificity, with the curve skewed toward the top‐left quadrant. The maximum Youden's Index was observed at a predicted probability cutoff of 0.573, yielding 100% sensitivity and 67% specificity. Using the coefficients from the logistic regression model, this probability threshold corresponds to a BED value of 1544.9 Gy_2.47_.

This suggests that patients treated with BED ≥ 1544.9 Gy_2.47_ were more likely to experience clinically meaningful pain relief, highlighting its potential as a planning parameter for GKRS in TN. Patients were stratified into tertile‐based BED groups (low ≤ 1544.9 Gy_2.47_, medium 1544.91–1872.9 Gy_2.47_, and high > 1872.9 Gy_2.47_) to assess whether categorical differences in dose were associated with clinical outcomes. However, neither one‐way ANOVA (*p* = 0.063) nor the Kruskal‐Wallis test (*p* = 0.09) demonstrated a statistically significant difference in BNI score improvement across these groups.

A repeated‐measures ANOVA was performed to evaluate the effects of BED on changes in BNI scores over time. While BED was modeled as a continuous covariate, the analysis did not yield valid *F* or *p*‐values, likely because the BED values lacked sufficient variability to support the interaction term.

### 3.4 BED and response in TRTN

**FIGURE 4:**
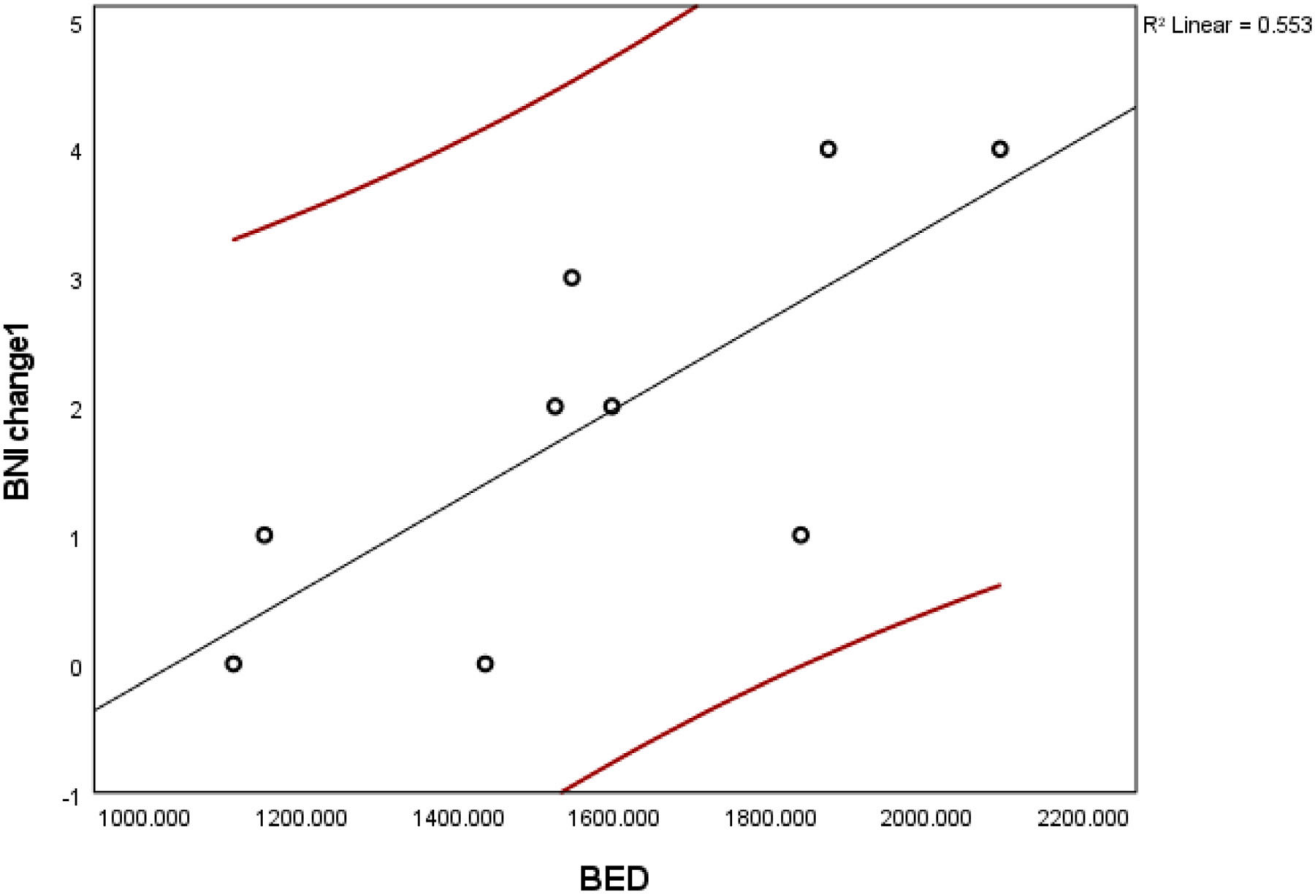
Scatterplot showing the relationship between biologically effective dose (BED) and Barrow Neurological Institute (BNI) score improvement at first follow‐up (BNI change 1) in patients with tumor‐related trigeminal neuralgia (TRTN). Each point represents an individual treatment session. The black regression line represents the best linear fit to the data, with red lines indicating the 95% confidence interval. A strong positive correlation was observed between BED and improvement in BNI score (R^2^ = 0.553), suggesting that higher BED values were associated with greater reductions in pain intensity. Some data points represent repeat Gamma Knife radiosurgery (GKRS) sessions for the same patient, consistent with the prime notation in Table 1.

**FIGURE 5:**
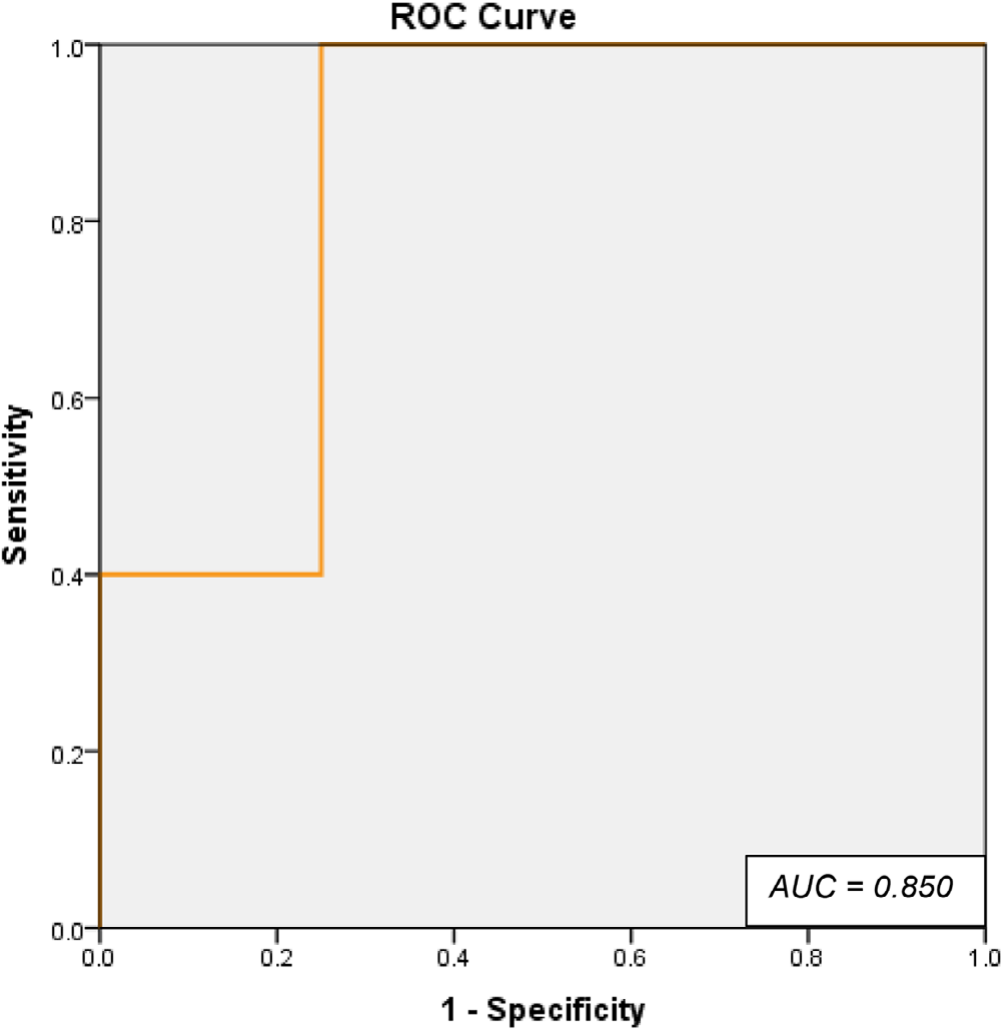
ROC curve illustrating the predictive performance of biologically effective dose (BED) for identifying responders (≥ 2‐point BNI score improvement at first follow‐up) among patients with tumor‐related trigeminal neuralgia (TRTN). The model yielded an AUC of 0.850, indicating excellent discriminative ability. The optimal BED threshold, determined using Youden's Index, corresponded to a predicted probability cutoff of 0.850 (BED = 1478.7 Gy_2_._47_), achieving 100% sensitivity and 75% specificity.

A focused subgroup analysis was conducted on the 10 treatment sessions in which TN was attributable to direct tumor compression. Of the 10 TRTN treatment sessions, nine had complete BED and clinical follow‐up data and were therefore included in the correlation and regression analyses shown in Figure 4. Detailed clinical, dosimetric, and outcome data for these sessions are presented in Table 2. One repeat session was excluded from the BED‐based analysis due to missing dosimetric information. Within this subset, BED remained strongly associated with BNI improvement at the first follow‐up, with Spearman's *ρ* = 0.797 (*p* = 0.01) and Pearson's *r* = 0.744 (*p* = 0.02). Similar to the full cohort, no significant associations were observed at subsequent follow‐ups. Binary logistic regression again demonstrated a borderline significant association between BED and responder status (*p* = 0.155), with excellent model discrimination (AUC = 0.850). The corresponding ROC curve (Figure 5) showed a maximum Youden's Index at a predicted probability cutoff of 0.750, yielding 100% sensitivity and 75% specificity. This cutoff mapped to a BED value of approximately 1478.7 Gy_2_._47_.

To further investigate categorical BED effects in this subgroup, patients were stratified into tertile‐based BED groups derived from the distribution of BED values: low (≤ 1463.8 Gy_2_._47_), medium (1463.8–1757.2 Gy_2_._47_), and high (> 1757.2 Gy_2_._47_). ANOVA revealed a statistically significant difference in BNI score change across these categories (*F*(2, 5.7) = 4.7, *p* = 0.05). These results suggest that higher BED levels were also associated with more substantial pain relief among patients whose TN was caused by direct tumor compression.

### 3.5 Facial numbness

Facial numbness was observed in 3 out of 12 patients (25%) during follow‐up. In all patients, the sensory changes were classified as BNI facial numbness score II, indicating mild hypoesthesia that was not bothersome to patients. Statistical analysis revealed no significant association between the development of facial numbness and any treatment parameters, including cumulative dose, maximum point dose, BED, or dose rate (*t*‐tests, all *p* > 0.05). Furthermore, changes in BNI pain scores across all three follow‐up visits were not significantly related to the occurrence of numbness.

### 3.6 GKRS for essential tremor

In addition to the TN cohort, two patients were identified who underwent GKRS for both ET and intracranial tumors, though the timing and approach differed. In the first patient, the tumor was treated initially, followed by a subsequent thalamotomy targeting the VIM 3 years later. In contrast, the second patient underwent a single‐session GKRS targeting both the intracranial tumor and the VIM for ET. This patient later received a second thalamotomy to treat contralateral tremor. All thalamotomies were delivered using a dose of 130 Gy, with BED values ranging from 1953.9 to 2111.2 Gy_2.47_. Both patients experienced functional improvement in tremor control without procedure‐related complications. A detailed summary of their treatment course and outcomes is presented in Table 3. Patient 1 showed continued tremor benefit at 5‐month follow‐up, while Patient 2 demonstrated benefit at 10 months after the first thalamotomy and at 2 months after the second thalamotomy, giving an overall follow‐up range of 2–10 months. The Fahn‐Tolosa‐Marin Tremor Rating Scale could not be assessed due to insufficient documentation in hospital records. However, all patients were under continuous neurological follow‐up for tremor management both before and after treatment.

**TABLE 3:** Clinical summary of two patients treated with Gamma Knife radiosurgery (GKRS) for essential tremor and coexisting intracranial tumors.

ID	Age (years)	Tumor type	Tumor location	GKRS targets	Thalamotomy side	Dose for ET (Gy)	Cumulative dose (Gy)	BED (Gy_2.47_)	Response summary	Complications
**1**	83	Vestibular schwannoma	Right IAC	Tumor (2015), VIM (2018)	Right	130	130	2111.2	Good tremor control at all follow‐ups	None
**2**	73	Meningioma	Right frontal	Tumor + Right VIM (Concurrent)	Right	120	120.1	1953.9	Marked improvement in left‐hand tremor	None
**2** ^*^	73	Meningioma	Right frontal	Left VIM	Left	130	130	2023.2	Improved right‐hand tremor; mild recurrence in left hand	Mild recurrence of left‐hand tremor

*Note*: One patient (Patient 2) underwent a second GKRS session for contralateral thalamotomy due to contralateral tremor. All thalamotomies were performed at a prescribed dose to 100% isodose line.

Abbreviations: BED, biologically effective dose; GKRS, Gamma Knife radiosurgery; VIM, ventral intermediate nucleus.

* Contralateral treatment in the same patient.

## 4 DISCUSSION

This retrospective analysis evaluated outcomes in patients undergoing GKRS for TN in the context of intracranial tumors, treated either simultaneously or in separate sessions. Despite analyzing multiple parameters, including prescribed dose, dose rate, and cumulative dose, no independent dosimetric predictors of pain relief or complication development were identified.

GKRS provided clinically meaningful relief in the majority sessions, with 76.5% of treatment sessions showing BNI score improvement, consistent with published rates of 70%–85%.^25^, ^32^, ^33^ Though not statistically significant, repeat treatments demonstrated numerically greater BNI improvement (mean change: 2.6 vs. 1.86), possibly due to an additive effect or higher baseline severity.

Interestingly, we observed that in patients who underwent repeat treatment, despite the cumulative radiation dose (mean 136 Gy), there was no statistically significant increase in pain relief or complications such as facial numbness. In addition, no radiation necrosis was observed in this series. These findings support the notion that, within controlled parameters, additional radiosurgical dosing may be delivered safely, although larger studies are needed to assess long‐term effects.

Among patients with TRTN, half of those with available follow‐up imaging showed no significant tumor shrinkage, and two of these required repeat GKRS for persistent pain. One of the radiological non‐responders had a vestibular schwannoma, a tumor type previously reported to be less responsive to radiosurgical size reduction in early follow‐up imaging.^34^ While limited by small numbers and lack of volumetric data, these findings suggest a potential link between tumor response and clinical pain relief that merits further study.

The analysis also evaluated the relationship between functional treatment parameters (dose to the TREZ, dose rate, and maximum point dose) and pain outcomes. No significant correlations were found between these factors and BNI score improvement at any follow‐up point. These results align with recent literature suggesting that optimal targeting and dose planning may be more influential than absolute dose values alone.^15^, ^35^ Facial numbness was infrequent, mild in severity, and did not appear to correlate with dosimetric parameters, consistent with prior observations in radiosurgical treatment for TN.^15^, ^16^, ^30^ However, radiation‐induced sensory effects can emerge over a longer latency interval, typically within 6–24 months post‐GKRS. Given that the mean follow‐up duration in our series was 9.3 months, it is possible that some delayed complications were not captured. This limitation should be considered when interpreting the low observed rate of facial numbness.

Figure 1 highlights the initial improvement in the patients' pain response, which later stabilizes, indicating a difference between the initial and long‐term pain response from SRS to TN. This aligns with previous studies of patients treated for idiopathic TN or TRTN, and the presence of any other intracranial tumor does not affect the pain response significantly.^16^, ^18^

The observed dependence of early pain relief on BED suggests that higher BED values were associated with greater initial clinical improvement. As illustrated in Figure 2, this suggests that BED may be a meaningful predictor of short‐term treatment response following GKRS. As the data distribution was confirmed to be normal, Pearson's correlation was considered valid and reported alongside Spearman's *ρ* for consistency across the manuscript. In contrast, the absence of statistically significant differences in BNI scores across tertile‐based BED groups emphasizes an important methodological consideration. While BED demonstrated a strong linear association with pain improvement when analyzed as a continuous variable, stratifying it into categorical groups may have decreased the analysis's sensitivity. This likely results from the limitations of a small sample size. This further supports the interpretation that individualized BED values, rather than arbitrary groupings, may be more informative when guiding treatment planning or predicting outcome.

Beyond its linear association with early BNI improvement, BED demonstrated potential clinical utility as a predictive marker. The optimal BED threshold identified through ROC analysis (1544.9 Gy) achieved high sensitivity (100%) and moderate specificity (67%) for predicting early responders. In the TRTN subgroup, a lower optimal threshold of 1478.7 Gy_2_․_47_ provided similarly high sensitivity (100%) and specificity (75%), reflecting a stronger and more uniform dose‐response relationship in anatomically driven TN. While this finding requires validation in larger cohorts, it suggests that a minimum BED threshold may be necessary to achieve durable pain relief. Clinically, this reinforces the importance of not only target coverage but ensuring that the delivered biological dose to the TREZ and adjacent nerve is sufficient to produce a meaningful pain response, especially in patients with complex presentations or prior subtherapeutic dosing. Incorporating BED into treatment planning could therefore help balance efficacy and safety, particularly when retreatment or overlapping dose fields are anticipated.

Our observed BED thresholds (1544.9 Gy_2_._47_ for the overall cohort and 1478.7 Gy_2_._47_ for TRTN) are broadly consistent with reports from larger idiopathic TN series. Tuleasca et al. identified effective BED ranges between 1550 and 2600 Gy_2_._47_, while Warnick et al. demonstrated that higher BED values were associated with improved pain relief and sensory outcomes in a multicenter cohort.^29^, ^35^ The fact that our thresholds lie at the lower end of these ranges may reflect the distinct radiobiological context of TRTN, in which direct mass effect on the nerve root leads to a more uniform response to radiosurgical dosing. Further studies with a larger cohort will clarify that relationship, and help guide treatments.

Among the subgroup of patients with TN secondary to direct tumor compression, several distinct patterns emerged that may help inform clinical management. First, BED showed a stronger and more statistically significant correlation with early BNI score improvement in TRTN group compared to the overall cohort. The Spearman's correlation coefficient for this group was *ρ* = 0.797 (*p* = 0.01), compared to *ρ* = 0.660 (*p* = 0.0054) in the full cohort, and the Pearson correlation similarly increased from *r* = 0.718 to *r* = 0.744. These findings suggest that in TRTN, the biological dose delivered to the TREZ may be a more sensitive and reliable predictor of early pain relief than in other forms of TN.

The stronger correlation between BED and pain relief observed in TRTN likely reflects the distinct pathophysiology of this subgroup. In these patients, pain is driven by direct mechanical distortion of the TREZ by the tumor. Radiosurgical dosing to the nerve in this context may therefore exert a more uniform radiobiological effect, resulting in a tighter dose–response relationship compared with idiopathic TN, where microvascular and neurochemical mechanisms introduce greater variability in outcomes.

This enhanced correlation may reflect the more direct mechanistic basis for pain in TRTN, namely, mass effect or distortion of the trigeminal nerve, which may respond more predictably to precise biological dosing. This contrasts with idiopathic TN, where microvascular or neurochemical pathophysiology may introduce greater variability in response. Importantly, the logistic regression model in the TRTN subgroup also showed improved predictive accuracy (AUC = 0.850 vs. 0.78 for the full cohort), with a lower optimal BED threshold (1367.51478.7 Gy_2_._47_ vs. 1544.9 Gy_2_._47_), further emphasizing the distinct dose‐response dynamics in this group.

Moreover, tertile‐based stratification of BED in the TRTN subgroup demonstrated a significant difference in BNI improvement across low, medium, and high BED categories, as confirmed by the ANOVA results, reinforcing the presence of a dose–response relationship. This was not observed in the broader cohort, reinforcing the idea that categorical BED stratification may be more meaningful in anatomically driven TN, where radiobiological effects are likely more uniform.

Taken together, these findings indicate that patients with TRTN may benefit from more dose‐tailored GKRS protocols, guided by BED metrics. They also suggest that dose escalation in TRTN may have higher predictive value and clinical payoff than in idiopathic TN. While larger, prospective studies are needed to validate these observations, the current data support the use of BED not only as a planning tool but as a biomarker of expected clinical benefit in TRTN.

Of particular note, our study included patients with TRTN as well as those with TN unrelated to the tumor but occurring concurrently. This reflects the heterogeneity of clinical presentations in functional neurosurgery and supports the practical value of individualized treatment strategies, especially when using a platform as flexible as stereotactic radiosurgery. A major benefit of GKRS is the capacity to deliver highly conformal plans, which leads to low‐dose tumor treatments contributing to the functional targets (TN or VIM), as observed in the low difference between the prescribed dose and cumulative dose of the first functional GKRS.

In addition to the TN cohort, we observed two patients who underwent GKRS for ET in the presence of intracranial tumors, further underscoring the versatility of radiosurgery in addressing coexisting functional and oncologic indications. Although not the primary focus of this analysis, the ET patients were included to highlight the feasibility and safety of treating multiple intracranial targets with precision. Both patients experienced meaningful tremor improvement, with one successfully undergoing a second thalamotomy for contralateral symptom control. Notably, no complications were observed in either patient despite cumulative dose exposures, and BED values remained within safe limits.

This study has several limitations. The small sample size and retrospective design limit the statistical power to detect subtle effects or uncommon complications and introduce potential selection bias related to patient eligibility and treatment planning decisions. Medication tapering post‐treatment was unstandardized and may have influenced recurrence patterns. Additionally, while BNI is widely accepted, it remains a subjective measure and may be confounded by hypoesthesia, especially when proximal targeting of the brainstem is involved. Objective outcome measures and standardized sensory testing should be incorporated in future studies. This study did not include formal volumetric analysis of tumor response; only qualitative imaging assessments were available. Follow‐up duration was also variable, which may have limited the detection of late recurrences or delayed toxicities. Prospective studies with standardized volumetric imaging and longer follow‐up are needed to better define these outcomes. Objective tremor rating scales, such as the Fahn–Tolosa–Marin Tremor Rating Scale, could not be applied due to a lack of documentation in clinical records. This limits the ability to quantify functional improvement in a standardized manner and is acknowledged as a limitation of this study.

While these ET patients represent a limited sample, they offer valuable insight into the potential for integrated radiosurgical approaches in selected patients. The ability to achieve symptom control without procedural morbidity reinforces the clinical utility of GKRS in managing complex presentations involving both tumor pathology and functional impairment. Future studies with larger cohorts and prospective designs will be essential to validate these findings and establish standardized protocols for multimodal radiosurgical interventions.

## 5 CONCLUSIONS

GKRS is a safe and effective treatment option for TN in the setting of coexisting intracranial tumors. While no single dosimetric factor consistently predicted clinical outcomes, the majority of patients experienced meaningful pain relief, and repeat treatment was well‐tolerated. BED emerged as a promising predictor of early treatment response, particularly in patients with TRTN, where its association with pain relief was stronger and more statistically robust. In this subgroup, a lower BED threshold (1478.7 Gy_2_._47_) demonstrated excellent discriminatory power for predicting responders, underscoring the potential value of BED in guiding dose planning for anatomically‐driven TN. For the broader cohort, a BED threshold of 1544.9 Gy_2_._47_ was identified, suggesting a minimum dose may be required to achieve optimal clinical effect. Additionally, the inclusion of patients treated for ET alongside intracranial tumors highlights the broader applicability of radiosurgery in managing complex cases involving both functional and oncologic targets. These findings support the potential of BED as a biologically informed planning parameter, particularly in TRTN group, and reinforce the versatility of GKRS as a treatment modality. Further prospective studies with larger cohorts are needed to validate these observations and support more individualized, dose‐guided radiosurgical strategies.

## AUTHOR CONTRIBUTIONS

**Sarthak Sinha**: Conceptualization; Data collection; statistical analysis; writing and review. **Victor Goulenko**: Conceptualization; data collection; statistical analysis; writing and review. **Venkatesh Shankar Madhugiri**: Writing and review. **Shefalika Prasad**: Data collection; writing and review. **Neil D. Almeida**: Writing and review. **Rohil Shekher**: Writing and review. **Matthew B. Podgorsak**: Writing and review. **Robert J. Plunkett**: Writing and review. **Dheerendra Prasad**: Conceptualization; writing and review.

## CONFLICT OF INTEREST STATEMENT

The author Dheerendra Prasad works as a consultant to Elekta AB. All the other authors have no conflicts of interest to declare.

## ETHICS STATEMENT

This study was performed in accordance with the Declaration of Helsinki. This human study was approved by the Institutional Review Board of Roswell Park Comprehensive Cancer Center. All adult participants provided written informed consent prior to participating in this study.

## Supporting information



Supporting Information

## Data Availability

The data that support the findings of this study are not publicly available due to privacy reasons but are available from the corresponding author upon request and IRB approval.
