# Supplementary material for: Treatment strategy in stereotactic radiosurgery for trigeminal neuralgia, essential tremor, and coexisting intracranial tumors: The impact of biologically effective dose on clinical outcome
Source: J Appl Clin Med Phys. 2025 Dec 29;27(1):e70436. doi: 10.1002/acm2.70436 (PMC12746042; doi:10.1002/acm2.70436)
Supplement: Supplementary file 1 — Supporting Information [file ACM2-27-e70436-s001.docx]

**Supplement 1**

(a)

$$BED= DT +\left( \frac{1}{\alpha/\beta} \right)\left[ \frac{\varphi(\Xi,\mu1)+c\varphi(\Xi,\mu2)}{1+c} \right]\left( DT \right)^{2}$$

(b) Where:

DT = Prescribed dose to 100% isodose line

c = 0.98;

φ(Ξ,μ1) = ((2/(0.00534836 x BOT))*(1-((1-EXP(-0.00534836 x BOT))/(0.00534836 x BOT)));

φ(Ξ,μ2) = (2/(0.0608024 x BOT)) x (1-((1-EXP(-0.0608024x BOT))/(0.0608024 x BOT)));

BOT = beam on time

(c) Example: DT = 80 Gy to 100% isodose line, and BOT of 38 minutes.

$BED= 80 +\left( \frac{1}{2.47} \right)\left[ \frac{0.93556093+(0.98 x 0.52814017)}{1+0.98} \right]\left( 80 \right)^{2}$ = 1981.62 Gy_2.47_
